# Impact of Comprehensive Breastfeeding Support Strategies on Exclusive Breastfeeding Rates at Discharge in a Neonatal Unit

**DOI:** 10.3390/nu18040575

**Published:** 2026-02-09

**Authors:** Alba Sánchez Ansede, Jorge Suances Hernández, Isabel María Fernández-Medina, Sara María Fernandez-Gonzalez, Alejandro Avila-Alvarez

**Affiliations:** 1 Neonatology Unit, Lactation Support Team, Pediatrics Departament, Complexo Hospitalario Universitario de A Coruña, 15006 A Coruña, Spain; 2 Research Support Unit, Complexo Hospitalario Universitario de A Coruña, 15006 A Coruña, Spain; jorge.suanzes.hernandez@sergas.es; 3 Department of Nursing, Physiotherapy and Medicine, University of Almería, 04120 Almeria, Spain; isabel.medina@ual.es; 4 Gastroenterology, Nutrition and Hepatology Unit, Pediatrics Department, Complexo Hospitalario Universitario de A Coruña, 15006 A Coruña, Spain; sara.maria.fernandez.gonzalez@sergas.es; 5 Grupo de Investigación en Pediatría y Neonatología, Instituto de Investigación Biomédica de A Coruña (INIBIC), 15006 A Coruña, Spain; 6 Neonatology Unit, Pediatrics Department, Complexo Hospitalario Universitario de A Coruña, 15006 A Coruña, Spain

**Keywords:** exclusive breastfeeding, neonatal unit, lactation support, pediatric nurse, lactation consultant, cohort study

## Abstract

**Background/Objectives:** Exclusive breastfeeding (EBF) at discharge from neonatal units is influenced by maternal, neonatal, and healthcare-related factors. Structured breastfeeding support may improve outcomes. This study aimed to assess the impact of specialized breastfeeding support provided by a pediatric nurse on EBF rates at discharge and to explore associated factors. **Methods:** A retrospective observational cohort study was conducted in a regional referral neonatal unit within the Spanish public healthcare system. Newborns admitted to the unit whose mothers intended to breastfeed were included. Two periods were compared: a pre-intervention period, April 2017–March 2019, (breastfeeding working group, written protocol, staff training, and donor human milk bank) and a post-intervention period, April 2019–March 2021, (incorporation of a lactation consultant and establishment of a Breastfeeding Committee). EBF at discharge and at 6 and 12 months was analyzed using descriptive statistics and multivariate logistic regression. **Results:** A total of 1136 newborns were included in the analysis. EBF at discharge increased from 39.6% in the pre-intervention period to 75.8% in the post-intervention period (*p* < 0.001). The post-intervention period was independently associated with EBF (OR 4.45; 95% CI: 3.45–5.75). Factors positively associated with EBF included participation in breastfeeding workshops, adequate milk expression frequency, initiation of breastfeeding at birth, and previous breastfeeding experience. Negative associations included hypogalactia, donor human milk use, and maternal pain. EBF rates were 38.5% at 6 months and 20.9% at 12 months. **Conclusions:** Specialized breastfeeding support within neonatal units was associated with a substantial increase in EBF at discharge, supporting its systematic integration into routine neonatal care.

## 1. Introduction

Breastfeeding (BF) provides significant benefits for newborns and mothers and is therefore considered the optimal form of nutrition in early life [1]. The World Health Organization (WHO) recommends initiating breastfeeding within the first hour of life, maintaining exclusive breastfeeding (EBF) for the first six months, and continuing breastfeeding alongside complementary feeding until at least two years of age or beyond [1].

Neonatal units represent a clinical environment in which the establishment and maintenance of BF may be compromised. Prematurity and other neonatal conditions, mother–infant separation, invasive procedures, and the organization of care itself have been identified as frequent barriers to the initiation and continuation of BF [2,3]. In addition, several studies indicate that mothers of preterm (PT) infants face additional challenges. Stress, immature sucking patterns, difficulties in advancing enteral feeding, complications related to prematurity, and prolonged hospitalization negatively affect EBF rates [4,5].

BF plays a particularly important role in PT and critically ill infants, in whom it may be considered a true therapeutic intervention [6,7]. Despite its well-established benefits, there remains substantial variability in how BF is promoted in neonatal units [8,9], reflecting the absence of a common policy and uniform strategies for its promotion. This heterogeneity hinders the proper establishment and maintenance of BF in neonatal units [10]. Several interventions have been shown to improve BF rates in neonatal units. Structured support programs led by healthcare professionals with specific breastfeeding training, standardized protocols, and active monitoring of milk production and feedings have been shown to significantly increase EBF at discharge [11,12,13]. In addition, skin-to-skin contact at birth and the kangaroo method from very early stages have been associated with a higher probability of initiating and maintaining BF, as well as higher rates of EBF at 6 and 12 months of age [14,15,16]. Other effective interventions include prioritizing the use of the mother’s own milk [17], supporting early and frequent milk expression [18], and ensuring access to human milk banks [19,20]. At the organizational level, the implementation of international standards such as the Initiative for the Humanization of Birth and Breastfeeding Care (IHAN/BFHI) and its adaptation to neonatal units (Neo-BFHI/Neo-IHAN) has been associated with improved breastfeeding support practices and early promotion of breastfeeding from admission [21,22].

Despite international recommendations to promote breastfeeding, EBF rates in neonatal units remain suboptimal. In developed countries, reported breastfeeding rates during NICU range from 13% to 49% [23,24], which is clearly lower than expected [25]. Consequently, it is necessary to analyze the impact of interventions focused on promoting breastfeeding, including the role of professional support provided by lactation consultants integrated into the healthcare team. Evaluating the effectiveness of these interventions and identifying factors associated with EBF will guide clinical decision-making and improve outcomes in neonatal units. Therefore, the objective of this study was to evaluate the impact of breastfeeding support interventions in a neonatal unit, specifically the support provided by a pediatric nurse with specialized training in breastfeeding, on EBF rates at discharge and to identify the factors associated with higher EBF rates.

## 2. Materials and Methods

A retrospective observational cohort study was conducted in the Neonatology Unit of a hospital within the Spanish public healthcare network, which serves as a regional referral center for an area with more than 5000 deliveries per year (2500 of which take place at the hospital itself). The unit provides care for newborns (NBs) with varying levels of clinical complexity and includes a Neonatal Intensive Care Unit (NICU) capable of delivering full critical care support. The unit follows structured protocols for the recording and follow-up of breastfeeding practices.

The study population included all newborns admitted during the study periods whose mothers expressed an intention to breastfeed and who remained under follow-up until 44 weeks of postconceptional age. Newborns whose mothers chose not to initiate breastfeeding were excluded. The study was approved by the corresponding Research Ethics Committee (approval code 2019/614).

Patients were grouped into two cohorts corresponding to two different care periods, distinguished by the availability of professional lactation counseling. The pre-intervention period (April 2017–March 2019) included the implementation of a unified breastfeeding protocol within the unit, staff training, the initiation of systematic data recording, and the opening of a donor human milk bank. The post-intervention period (April 2019–March 2021) was characterized by the incorporation of a lactation consultant and the establishment of a multidisciplinary Breastfeeding Committee within the hospital. These periods are hereafter referred to as the pre-counseling and post-counseling periods, respectively. Comparison between both periods were performed, along with an evaluation of additional variables potentially associated with exclusive breastfeeding. The post-intervention cohort was sub-divided into two groups (before and after March 2020) to assess potential influence of COVID pandemic in breastfeeding rates.

The primary outcome was the rate of exclusive breastfeeding at discharge. Secondary outcomes included exclusive breastfeeding rates at 6 months and at 12 months of age. Covariates included maternal and neonatal characteristics, perinatal factors, clinical course during hospitalization, and routinely recorded feeding practices. Maternal pain was defined as breastfeeding-related pain, including nipple pain and breast pain occurring during direct breastfeeding or milk expression, as documented by the lactation consultant during hospitalization. Postpartum pain unrelated to breastfeeding and chronic pain conditions was not included. Hypogalactia was defined based on the Academy of Breastfeeding Medicine as the insufficient production of breast milk to meet an infant’s nutritional requirements.

Data were obtained from the electronic medical record and the breastfeeding chart used in routine clinical practice. All information was recorded in a data collection form specifically designed for this study.

Data were exported and analyzed using IBM SPSS Statistics software, version 22.0 (IBM Corp., Armonk, NY, USA). Categorical variables were described using absolute frequencies and percentages, and continuous variables using mean and standard deviation (SD). Figures were generated or refined using generative artificial intelligence tools (ChatGPT v 5.2; OpenAI).

Bivariate analyses were performed to compare the pre-counseling and post-counseling periods. Categorical variables were analyzed using Pearson’s chi-square test, allowing up to 25% of cells with expected frequencies below 5. Continuous variables were compared using Student’s *t*-test or the Mann–Whitney U test when the assumption of normality was not met. Prior to model construction, multicollinearity among candidate variables was assessed using variance inflation factors (VIFs). Variables showing significant collinearity were excluded from the final model to ensure estimate stability.

Finally, a binary logistic regression model was constructed to identify factors associated with exclusive breastfeeding at discharge. Results were expressed as odds ratios (ORs) with 95% confidence intervals (CIs), adjusted for assignment to the pre- or post-counseling group. A *p* value < 0.05 was considered statistically significant.

## 3. Results

A total of 1136 NBs were included in the analysis, of whom 54% (n = 613) belonged to the pre-counseling period and 46% (n = 523) to the post-counseling period.

The mean gestational age was 262.10 days (SD = 27.05), equivalent to 37 + 4 weeks of gestation. Mean birth weight was 2795.63 g (SD = 861.56). A total of 58.3% (n = 662) were male, and 93.1% (n = 1058) were born at the study hospital. Admission to the Neonatal Intensive Care Unit (NICU) was required for 32% of NBs (n = 363), and 27.5% (n = 312) required resuscitation at birth. The most frequent neonatal diagnoses were prematurity (22.6%, n = 257), risk of infection/sepsis (33.8%, n = 384), and respiratory disease (6.9%, n = 78).

Mean days of hospitalization were 11.59 (SD = 17.33). Enteral feeding was initiated early, at a mean of 0.56 days of life (SD = 1.43), while oral feeding was initiated at a mean of 3.25 days of life (SD = 9.11). The mean length of hospital stay was 11.59 days (SD = 17.33).

The mean maternal age was 33.78 years (SD = 5.79). Nearly half of the mothers were primiparous (49.7%, n = 565), while 50.3% (n = 571) had previous children. Only 13.1% (n = 149) reported prior breastfeeding experience.

A total of 61.9% (n = 703) of deliveries were vaginal, and 38.1% (n = 433) were by cesarean section, mainly due to suspected fetal compromise. Multiple pregnancies accounted for 5.8% (n = 66) of cases. Among maternal comorbidities, hypothyroidism (5.8%, n = 66) and diabetes (5.6%, n = 64) were the most frequent. Baseline characteristics were comparable between pre and post-intervention groups (Supplementary Table S1).

Initial feeding after birth consisted of breastfeeding in 52.7% (n = 599), infant formula in 40.6% (n = 461), and donor human milk in 5.6% (n = 64). During hospitalization, 97.2% of NBs (n = 1104) received some form of human milk (direct breastfeeding, expressed mother’s milk, or donor milk). However, 59.4% (n = 675) required infant formula at some point, and 7% (n = 79) received donor human milk. Milk expression was performed in 75.6% of cases (n = 859), of which 51.7% (n = 587) used mechanical expression and 23.5% (n = 267) combined mechanical and manual techniques. An extraction frequency considered optimal was achieved by 56.5% (n = 642) of mothers.

Regarding direct breastfeeding, 89.7% (n = 1019) of newborns were placed at the breast at some point. However, sucking was assessed as ineffective in 52.7% (n = 599) of cases, mainly due to oral dysfunction (primary or secondary) and the clinical condition of the newborn. Postural breastfeeding support measures were widely implemented (72.8%, n = 827), representing the most frequent intervention and reflecting the high prevalence of ineffective sucking. A total of 61.9% (n = 703) of NBs received breastfeeding support from a pediatric nurse specialized in breastfeeding, 49.8% (n = 566) required specific supportive practices (breast compression and nipple shield use), and 20.9% (n = 237) used donor human milk at some point during the process.

**Figure 1:**
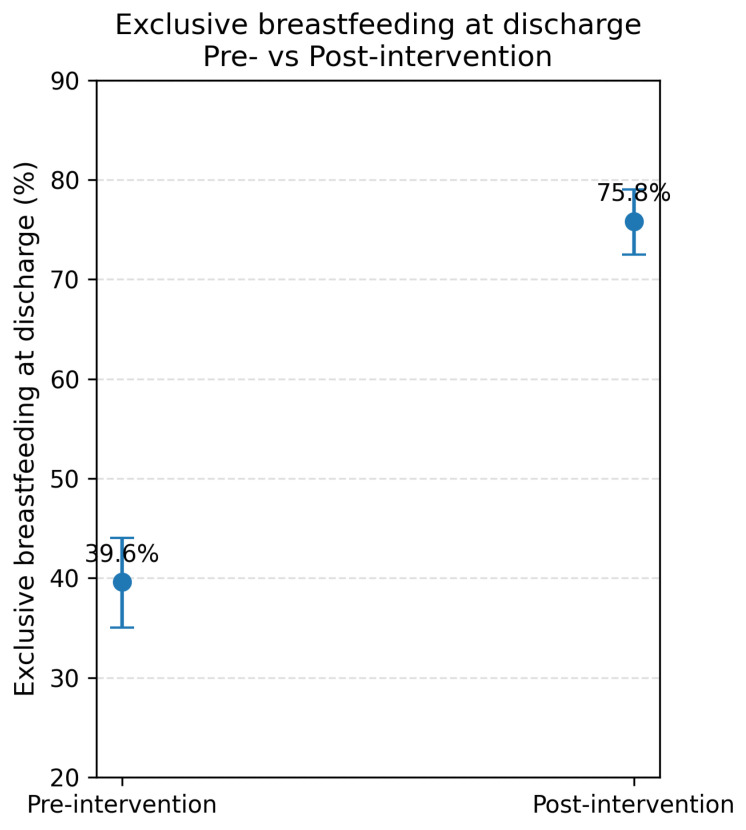
Percentage of exclusive breastfeeding at hospital discharge before and after the intervention. Blue dots represent mean values, and error bars denote 95% confidence intervals (95% CI).

Exclusive breastfeeding rates at discharge differed between cohorts. During the pre-intervention period, exclusive breastfeeding was achieved in 39.6% (n = 243), whereas in the post-intervention period it increased to 75.8% (n = 396) (*p* < 0.001). See Figure 1.

The probability of exclusive breastfeeding at discharge was significantly higher during the post-intervention period (75.8% vs. 39.6%, *p* < 0.001). Unadjusted analysis showed that care during the post-counseling period increased the likelihood of achieving exclusive breastfeeding by more than fourfold (OR 4.45; 95% CI: 3.45–5.75). At follow-up, exclusive breastfeeding was maintained in 38.5% of infants at 6 months and in 20.9% at 12 months of age. There were no differences in the rate of exclusive breastfeeding among patients in the pre-pandemic and post-pandemic period (72.3 vs. 79.1%, respectively, *p* value 0.112).

In multivariate analysis, attendance at breastfeeding workshops delivered by the pediatric nurse with specialized training in breastfeeding was the strongest predictor of exclusive breastfeeding at discharge, substantially increasing the odds of achieving it (OR 19.05; 95% CI: 3.81–95.22). Optimal frequency of maternal milk expression during hospitalization was also associated with higher exclusive breastfeeding rates (OR 4.89; 95% CI: 2.78–8.60), as was initiation of feeding with breastfeeding immediately after birth (OR 3.83; 95% CI: 1.10–13.37). Other factors positively associated with exclusive breastfeeding included previous breastfeeding experience (OR 3.14; 95% CI: 1.58–6.27) and the application of breastfeeding-supportive practices (breast compression and nipple shield use) following assessment by the lactation consultant (OR 2.11; 95% CI: 1.33–3.36).

Conversely, hypogalactia was associated with a significantly lower likelihood of achieving exclusive breastfeeding (OR 0.003; 95% CI: 0.000–0.022). Use of donor human milk was also associated with lower rates of exclusivity (OR 0.21; 95% CI: 0.08–0.52), as was the presence of maternal pain during breastfeeding (OR 0.37; 95% CI: 0.25–0.56).

**Figure 2:**
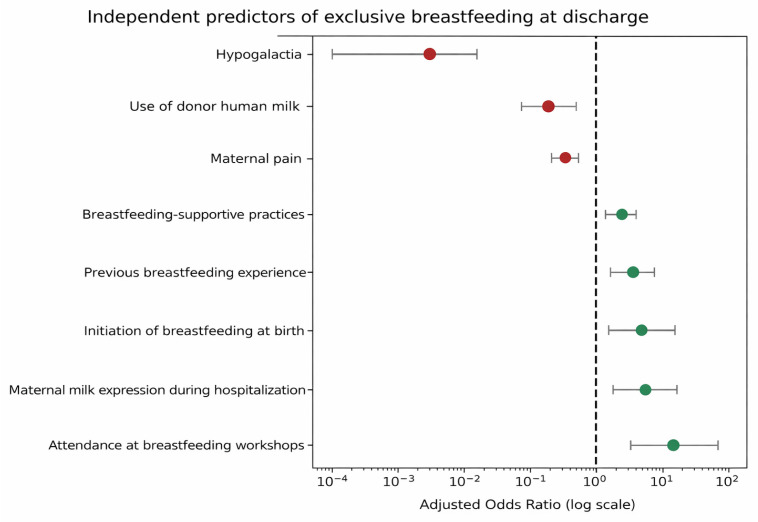
Multivariate logistic regression analysis of factors associated with exclusive breastfeeding at hospital discharge. Green and red dots represent adjusted odds ratios (ORs), horizontal lines indicate the corresponding 95% confidence intervals (95% CI), and the dashed vertical line represents the null value (OR = 1).

The complete results of the multivariate logistic regression model, including adjusted odds ratios and 95% confidence intervals, are presented in Figure 2.

## 4. Discussion

The protection and promotion of BF constitute an essential component of modern neonatal care. This study, conducted in a cohort of more than 1000 neonates admitted to a neonatal unit, demonstrates that structured breastfeeding support interventions, including specialized professional support, group-based education, and optimization of milk expression, are associated with a higher likelihood of establishing EBF at discharge. These findings confirm the need to incorporate lactation support services into the clinical teams of neonatal units.

The impact of lactation counseling observed in our study is comparable to that reported for interventions based on the Neo-BFHI. Song et al. (2023) [17], reported a significant increase in EBF rates among preterm newborns (PTNs) (10–25%) following the implementation of Neo-BFHI strategies in NICUs [17], whereas in our study the observed increases nearly doubled this magnitude. This difference suggests that the applied model, integrating continuous staff training, standardization of practices, and expert clinical support, may generate a more powerful synergistic effect than single-component or partially implemented interventions. Available evidence indicates that breastfeeding programs with the greatest impact are those that simultaneously combine multiple intervention domains [25,26], which is fully consistent with our approach and may explain the superior outcomes observed.

Comparison with studies focused on PTNs further supports this interpretation. In 2016, both Briere et al. [14] and Ericson et al. [23] reported EBF rates at discharge of approximately 50–60% after implementing breastfeeding support interventions primarily based on maternal education, promotion of early contact, and strategies to transition to direct breastfeeding. Although these programs demonstrated benefits, the increases were smaller than those observed in our cohort, suggesting that more comprehensive interventions, such as the incorporation of a pediatric nurse specialized in breastfeeding, practice standardization, and intensive clinical support, may achieve a greater impact.

The role of specialized breastfeeding support has been widely documented. Mercado et al. showed that the presence of lactation consultants significantly increases the availability of mothers’ own milk and direct breastfeeding in very low birth weight infants, whereas the absence of expert counseling limits the effectiveness of other structural interventions [27]. Although their study did not directly assess EBF, it provides clear evidence that specialized clinical support acts as a multiplier that enhances the effects of other strategies, a pattern also observed in our cohort.

Mothers of PTNs or infants with complex conditions face additional challenges, including mother–infant separation, immature sucking patterns, and clinical instability, which require intensive support. Qualitative studies have highlighted the importance of specialized interventions to overcome these barriers [28]. Moreover, staff training has been shown to significantly improve EBF practices in high-risk neonates [29]. Our findings are consistent with this evidence, reinforcing the notion that the quality of clinical support is a central determinant of maximizing EBF.

EBF rates at 6 months (38.5%) and at 12 months (20.9%) in our cohort fall within the range reported in the scientific literature for PTNs [30]. Ericson et al. (2018) observed that only 23% of infants maintained EBF at 6 months and 21% received any breastfeeding at 12 months [31]. Despite the expected post-discharge decline in this population, our results exceed those reported in other studies, suggesting a potential protective effect of the interventions implemented during hospitalization. This finding further underscores the need to develop post-discharge strategies, such as dedicated follow-up lactation consultations, to reinforce breastfeeding continuation.

The strongest predictor identified in the multivariate analysis was participation in workshops led by the pediatric nurse specialized in breastfeeding, in line with reviews indicating that educational interventions incorporating guided practice and skills training are particularly effective for mothers of preterm infants [31,32,33]. Nevertheless, early and frequent milk expression and early initiation of breastfeeding were also associated with higher odds of EBF, consistent with studies identifying early expression as a key determinant of milk production and lactogenesis II [19,34]. Previous breastfeeding experience significantly increased the likelihood of success, a finding consistent with self-efficacy models described in the literature. Ericson et al. (2018) [23], reported that mothers with prior breastfeeding experience tend to demonstrate greater confidence, higher tolerance of initial difficulties, and a more positive perception of their ability to produce milk and sustain breastfeeding in complex situations such as neonatal hospitalization [31].

Among negative factors, hypogalactia emerged as the most critical barrier to achieving EBF, replicating findings in the literature where low milk supply has repeatedly been identified as a key determinant of early breastfeeding discontinuation in neonatal settings. Both Dong et al. (2022) [35] and Karacan et al. (2025) [36] have shown that perceived low milk supply and objectively insufficient volumes generate a negative feedback loop characterized by increased maternal anxiety, reduced expression frequency, and compromised breastfeeding stability [35,36].

The use of donor human milk, although essential from a nutritional and safety perspective for PTNs or clinically unstable infants, often acts as an indirect marker of difficulties in producing mothers’ own milk. According to the review by William et al. [37], its use does not increase the likelihood of EBF at discharge and may, in some cases, reflect underlying challenges that require more intensive support [37]. This consistency with existing evidence suggests that donor milk should be interpreted as a supportive measure rather than a direct facilitator of exclusivity, reinforcing the importance of targeted interventions aimed at preserving and stimulating maternal milk production. Moreover, the use of donor human milk may also be interpreted as a marker of underlying clinical complexity or early breastfeeding difficulties, and not as a causal factor reducing exclusive breastfeeding.

Maternal breastfeeding-related pain was also negatively associated with exclusive breastfeeding at discharge. In this study, pain referred specifically to nipple and breast pain occurring during breastfeeding or milk expression, and not to postpartum pain unrelated to feeding or chronic pain conditions. Studies such as that by Boucher et al. [38], have demonstrated that nipple trauma, hypersensitivity, and discomfort associated with milk expression constitute critical obstacles to breastfeeding maintenance in neonatal settings [38]. Breastfeeding-related pain may interfere with effective latch and milk transfer, reduce adherence to frequent milk expression, and negatively impact maternal confidence, thereby increasing the risk of early breastfeeding discontinuation. These factors may interfere with both technique and adherence to frequent expression regimens, compromising process efficiency, so early identification and targeted management of breastfeeding-related pain through individualized technical support, postural adjustments, and latch optimization in neonatal units are important. In this regard, several studies highlight that early intervention by speech therapists and physiotherapists specialized in oral function can improve suck–swallow coordination, optimize milk transfer, and reduce maternal pain, thereby facilitating the transition to more effective and sustainable breastfeeding [39].

Finally, postural measures and breastfeeding-supportive techniques, including breast compression, latch adjustment, and structured use of specific positions, were positively associated with EBF. This observation is consistent with studies emphasizing the role of appropriate positioning in achieving effective latch, reducing pain, and improving milk transfer efficiency, key elements for consolidating breastfeeding, particularly in neonates with oral immaturity or anatomical difficulties [40,41]. Taken together, these findings reinforce the importance of a multidisciplinary approach that combines clinical management, technical support, and expert guidance from the very beginning of the breastfeeding process.

The magnitude and consistency of the observed increase in exclusive breastfeeding at discharge support an interpretation that extends beyond a purely organizational association, pointing to a substantial transformation in the breastfeeding care model within the neonatal unit rather than the effect of an isolated structural change. Taken together, these findings suggest a shift toward a more continuous, competence-oriented, and mother-centered approach to breastfeeding support, characterized by greater continuity of care, enhanced maternal skill acquisition, and sustained professional engagement, while acknowledging the observational nature of the study and its inherent limitations.

This study has several limitations that should be considered when interpreting the results. The observational design does not allow causal relationships to be established between breastfeeding support interventions and EBF. As this was a before–after study, the possibility of temporal confounding cannot be excluded, since organizational, cultural, or clinical practice changes other than the introduction of the lactation consultant may have occurred during the post-intervention period and could have influenced breastfeeding outcomes. Nevertheless, the magnitude of the observed associations reinforces their clinical relevance.

In addition, the study was conducted in a single neonatal unit, and therefore the generalizability of the findings to other settings should be approached with caution. However, the homogeneity of neonatal care, the prospective design, and the large sample size with comprehensive variable collection represent important strengths of this study.

## 5. Conclusions

The implementation of a comprehensive breastfeeding support model was associated with a substantial increase in exclusive breastfeeding rates at discharge. Group-based education, optimization of milk expression, and specialized support provided by a pediatric nurse with specific training in breastfeeding emerged as key elements in facilitating the establishment of breastfeeding in this neonatal population. In contrast, hypogalactia, maternal pain, and the need for supplementation with donor human milk were identified as relevant barriers that require timely detection and appropriate clinical management. These findings reinforce the importance of integrating practices aligned with Neo-BFHI/Neo-IHAN standards and strengthening the role of expert breastfeeding support to improve outcomes in neonatal units.

## Data Availability

The data that support the findings of this study are available from the corresponding author, A.S.A., upon reasonable request.

## Supplementary Materials

The following supporting information can be downloaded at: https://www.mdpi.com/article/10.3390/nu18040575/s1, Table S1: Baseline characteristics and outcomes before and after the intervention.

## Abbreviations

The following abbreviations are used in this manuscript:

BF	Breastfeeding
EBF	Exclusive Breastfeeding
NBs	Newborns
NICU	Neonatal Intensive Care Unit
PT	Preterm Newborn
WHO	World Health Organization
BFHI	Baby-Friendly Hospital Initiative
Neo-BFHI	Baby-Friendly Hospital Initiative for Neonatal Units
ORs	Odds Ratios
CI	Confidence Interval
SD	Standard Deviation
