# Supplementary material for: Impact of Comprehensive Breastfeeding Support Strategies on Exclusive Breastfeeding Rates at Discharge in a Neonatal Unit
Source: Nutrients. 2026 Feb 9;18(4):575. doi: 10.3390/nu18040575 (PMC12943618; doi:10.3390/nu18040575)
Supplement: Supplementary file 1 [file nutrients-18-00575-s001.zip › nutrients-4129519-supplementary.pdf]

Table S1. Baseline characteristics and outcomes before and after the intervention

A) Quantitative Variables

Variable	Pre n	Mean (SD)	Median (IQR)	Post n	Mean (SD)	Median (IQR)	p value
Gestational age (days)	614	262.8 (24.4)	271 (249–281)	522	262.2 (25.1)	270.5 (249–281)	0.760
Birth weight (g)	614	2777.6 (847.2)	2925 (2170–3420)	522	2816.9 (878.4)	2960 (2150–3480)	0.457
Admission weight (g)	614	2767.9 (846.4)	2900 (2170–3400)	522	2807.3 (877.6)	2945 (2150–3470)	0.443
Length of hospital stay (days)	611	12.4 (17.2)	6 (2–15)	520	10.6 (17.4)	4 (2–10)	0.003
Previous breastfeeding (n)	59	1.1 (0.3)	1,0 (1,0 - 1,0)	69	1.2 (0.5)	1,0 (1,0 - 1,0)	0.442

B) Qualitative Variables

Variable	Pre n (%)	Post n (%)	P value
Sex (male)	356 (58.1%)	306 (58.6%)	0.853
Cesarean section	240 (39.2%)	192 (36.9%)	0.414
Maternal comorbidity	105 (17.1%)	126 (24.2%)	0.122
Prematurity	129 (21.0%)	128 (24.5%	0.872
Multiple gestation	66 (10.7%)	42 (8.1%)	0.129
Primipara	321 (52.3%	244 (46.7%)	0.161
NICU	193 (31.4%)	171 (32.8%)	0.633

Abbreviations: SD, standard deviation; IQR, interquartile range; CI, confidence interval; NICU, neonatal intensive care unit
